# Estimating Risk of Elder Abuse: Intergenerational Communication/Empathy, Census-Based Assessment

**DOI:** 10.1093/geroni/igaa057.3213

**Published:** 2020-12-16

**Authors:** Heng Wu, Lyn Holley

**Affiliations:** The University of Nebraska at Omaha, Omaha, Nebraska, United States

## Abstract

COVID highlights the importance of accurate estimates of the risk of elder abuse to guide prevention. Reliability of data is problematic; reports have issues with consistency of definitions, time periods, and stigma. This paper demonstrates the use of US Census data to estimate risk of elder abuse by mapping generational incongruities between care-givers and care-receivers that invite dissonance (Cohen, 2011). Using the 2014-2018 Nebraska Public Use Microdata and 5-year American Community Survey, this research identifies and profiles personal care aides/nursing aides for institutionalized persons (65+). Data reveal generation gaps in age, education, and race between care-receivers and care-givers that are geographically comparable in the three most populous counties (the Big-3), but different for the 90 rural counties. In Nebraska’s Big-3 Counties the difference in education between care-givers (ages 25+) and care-receivers (65+) is considerable; 42.9% of Big-3 care-givers have some college, while 43.6% of care-receivers have only high school. Intergenerational differences in education are greater in Nebraska’s 90 rural counties than in the Big-3; 41.2% of rural care-givers have some college; 46.1% of rural care-receivers have only high school. Racial intergenerational differences are greater in Big-3 than in rural counties; 90.3% of care-receivers and only 62.3% of care-givers are non-Hispanic White. For rural Nebraskans, intergenerational differences in race are smaller, 97.2% of care-receivers and 79.4% of care-givers are non-Hispanic White. This type of analysis can be used to identify geographic settings where elder abuse is most likely to happen and guide the development of preventive measures that mitigate elder abuse.

